# p38 regulates the tumor suppressor PDCD4 via the TSC-mTORC1 pathway

**DOI:** 10.15698/cst2021.12.260

**Published:** 2021-11-23

**Authors:** Clarissa Braun, Karl Katholnig, Christopher Kaltenecker, Monika Linke, Nyamdelger Sukhbaatar, Markus Hengstschläger, Thomas Weichhart

**Affiliations:** 1 Center of Pathobiochemistry and Genetics, Institute of Medical Genetics, Medical University of Vienna, Vienna, Austria.; 2 Clinical Division of Endocrinology and Metabolism, Department of Internal Medicine III, Medical University of Vienna, Vienna, Austria.; 3 Department of Internal Medicine III, Division of Nephrology and Dialysis, Medical University of Vienna, Vienna, Austria.

**Keywords:** macrophages, cancer, rapamycin, MK2, PDCD4, p38, mTORC1, TSC1, TSC2

## Abstract

Programmed cell death protein 4 (PDCD4) exerts critical functions as tumor suppressor and in immune cells to regulate inflammatory processes. The phosphoinositide 3-kinase (PI3K) promotes degradation of PDCD4 via mammalian target of rapamycin complex 1 (mTORC1). However, additional pathways that may regulate PDCD4 expression are largely ill-defined. In this study, we have found that activation of the mitogen-activated protein kinase p38 promoted degradation of PDCD4 in macrophages and fibroblasts. Mechanistically, we identified a pathway from p38 and its substrate MAP kinase-activated protein kinase 2 (MK2) to the tuberous sclerosis complex (TSC) to regulate mTORC1-dependent degradation of PDCD4. Moreover, we provide evidence that TSC1 and TSC2 regulate PDCD4 expression via an additional mechanism independent of mTORC1. These novel data extend our knowledge of how PDCD4 expression is regulated by stress- and nutrient-sensing pathways.

## INTRODUCTION

Programmed cell death protein 4 (PDCD4) is an RNA-binding tumor suppressor protein that is vital for inhibiting carcinogenesis, tumor progression and invasion [1]. Low PDCD4 expression promotes neoplastic transformation [2]. The activity of the PDCD4 protein seems to be mainly determined by its stabilization [3].

Recent data showed that PDCD4 is also a modifier of inflammatory processes in macrophages [4–7]. Cellular PDCD4 levels remain stable throughout the process of monocyte/macrophage differentiation [8], but are upregulated upon starvation or induction of apoptosis [9]. Interestingly, macrophages reduce PDCD4 expression in cancer cells by mTOR-mediated proteasomal degradation [10]. In contrast to starvation, mitogenic signals such as growth factors or pathogen-associated molecules such as lipopolysaccharide (LPS) lead to the ubiquitination of PDCD4 by F-box/WD repeat-containing protein 1A (βTRCP) ubiquitin ligases and its subsequent degradation by the proteasome [11]. Mechanistically, mitogenic signals activate the phosphoinositide 3-kinase (PI3K)-mammalian target of rapamycin complex 1 (mTORC1) pathway. mTORC1 then phosphorylates its substrate ribosomal protein S6 kinase beta-1 (S6K1), which directly phosphorylates PDCD4 as trigger for its ubiquitination and degradation [11]. Inhibition of mTORC1 with rapamycin prevents degradation of PDCD4 [5].

Whether other signal transduction pathways in addition to PI3K regulate PDCD4 expression via mTORC1 is largely unknown. We and others have previously found that the mitogen-activated kinase (MAPK) p38α contributes to the activation of mTORC1 [12, 13]. Specifically, the p38 substrate MAP kinase-activated protein kinase 2 (MK2) phosphorylates Ser1210 on the tuberous sclerosis complex 2 (TSC2, Tuberin), a negative regulator of mTORC1 signaling, and contributes to inflammatory cytokine expression in macrophages [12]. In the current study, we wanted to investigate whether p38 controls PDCD4 expression.

## RESULTS

### p38 negatively regulates PDCD4

**Figure 1:**
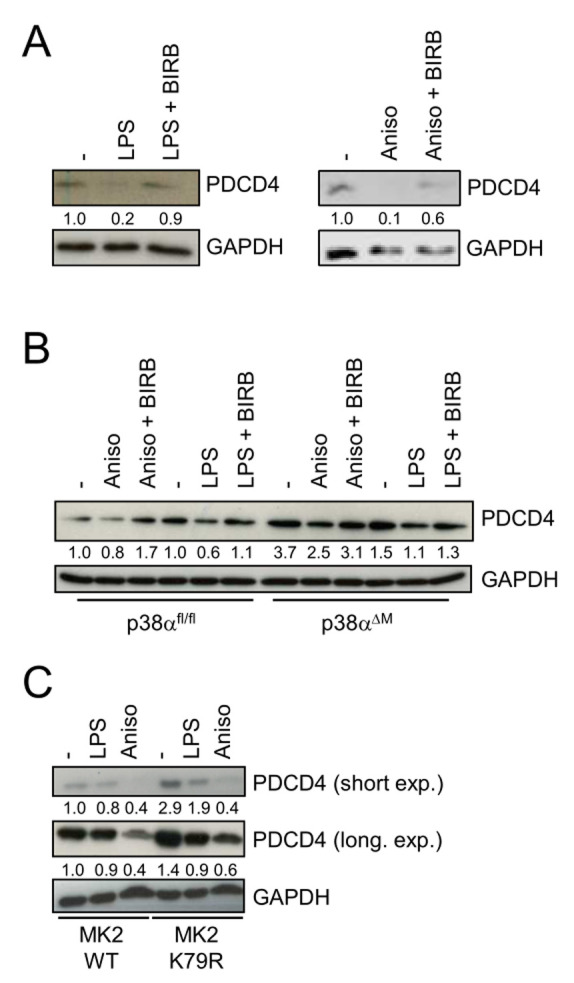
FIGURE 1: p38 promotes PDCD4 degradation. **(A)** Bone marrow-derived macrophages (BMDMs) were treated with BIRB796 (BIRB) as depicted and then stimulated with LPS or anisomycin (Aniso) only for 4 hours. **(B)** *p38*^*f*l/fl^ and *p38*^αΔM^ BMDMs were stimulated with Aniso or LPS for 4 hours. **(C)** *Mk2*^−/−^ macrophages reconstituted with either MK2 K79M mutant or WT MK2 were stimulated with Aniso or LPS for 4 hours. Cell lysates were analyzed by immunoblotting.

To study a potential role of p38 on PDCD4, we used the two well-known p38 activators anisomycin and LPS. Anisomycin has an inhibitory effect on protein translation [14], whereas LPS stimulates inflammatory protein synthesis. In bone marrow-derived macrophages (BMDMs) we found that LPS and anisomycin induced the reduction of PDCD4 (**Fig. 1A**). Interestingly, chemical inhibition of p38 with BIRB796 [15] prevented the LPS- or anisomycin-induced decrease of PDCD4 (**Fig. 1A**). To genetically corroborate these findings, we analyzed p38α-deficient BMDMs. We detected higher levels of PDCD4 in unstimulated p38α-deficient BMDMs compared to their control cells (**Fig. 1B**). Of note, PDCD4 was still partially lost in LPS- or anisomycin-stimulated p38α-deficient cells. Moreover, levels of PDCD4 were increased in a macrophage cell line that expressed a catalytic dead mutant of MK2 (K79R) to prevent p38-mediated phosphorylation and activation (**Fig. 1C**). These data suggest that p38 and its substrate MK2 negatively regulate the expression of PDCD4 in macrophages.

### p38 controls PDCD4 via TSC1/TSC2

**Figure 2:**
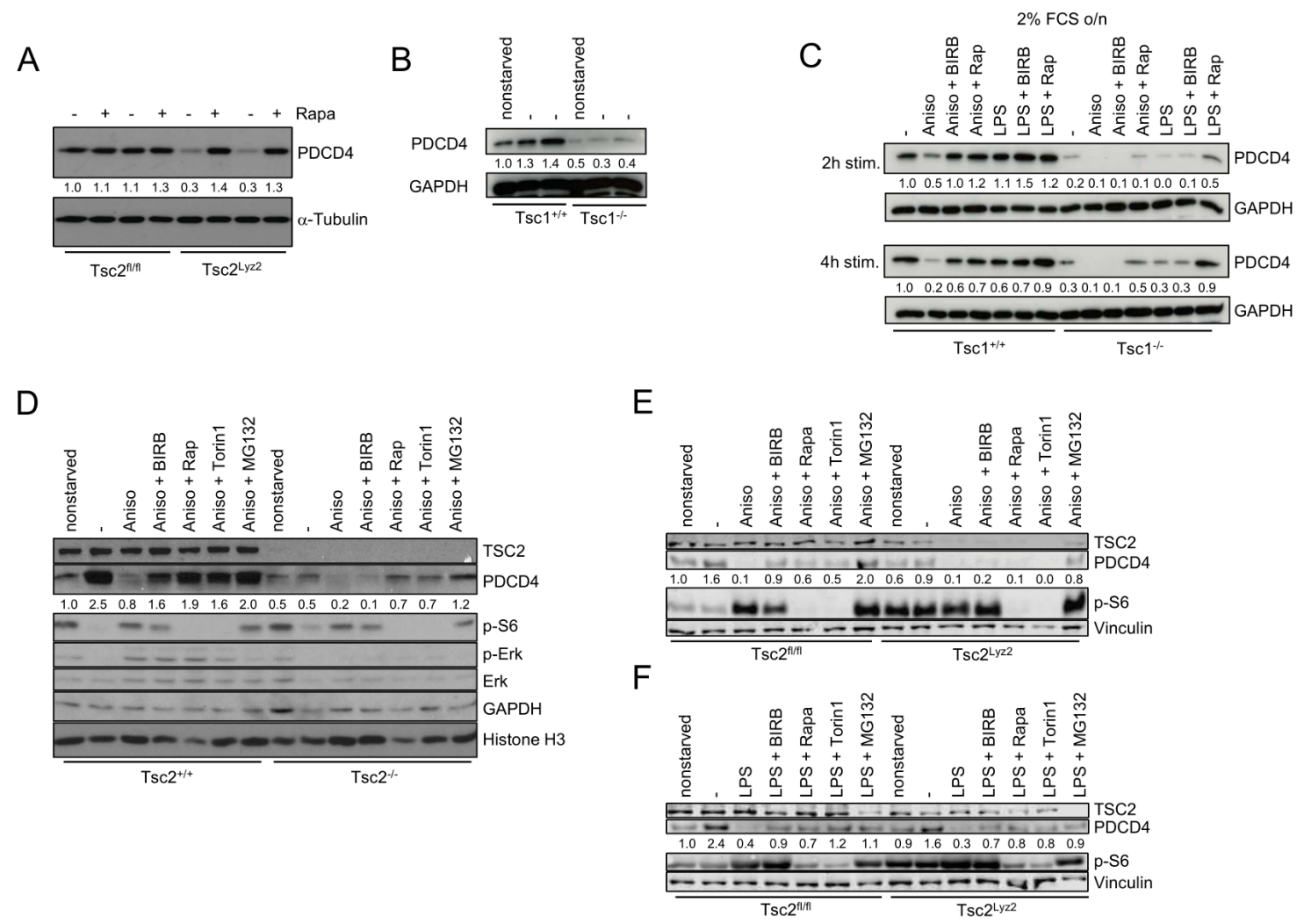
FIGURE 2: The TSC complex links p38 to PDCD4. **(A)** BMDMs from *Tsc2*^fl/fl^ and *Tsc2*^Lyz2^ were cultivated with or without rapamycin for 12 hours. **(B)** *Tsc1*^+/+^ and *Tsc1*^−/−^ fibroblasts were non-starved or serum-starved as indicated and subsequently, cell lysates were prepared. **(C)** *Tsc1*^+/+^ and *Tsc1*^−/−^ fibroblasts were serum-starved overnight. Afterwards, cells were treated with the indicated inhibitors for 90 minutes followed by Aniso stimulation for 2 and 4 hours, respectively. **(D)** *Tsc2*^+/+^ and *Tsc2*^−/−^ fibroblasts were non-starved where indicated or were serum-starved overnight. Inhibitor treatment of cells for 90 minutes preceded 4 hours of Aniso treatment. Cell lysates were analyzed by immunoblotting. **(E, F)** BMDMs from *Tsc2*^fl/fl^ and *Tsc2*^Lyz2^ mice were pretreated with the inhibitors BIRB796, rapamycin, Torin 1 or MG-132 for 90 minutes and subsequently elicited with LPS or Aniso for 4 hours.

The complex of TSC1 (Hamartin) and TSC2 is a major negative regulator of mTORC1, and its involvement in mTORC1-mediated degradation of PDCD4 has been recently suggested [16]. Indeed, deletion of TSC2 in BMDMs strongly abrogated expression of PDCD4 (**Fig. 2A**). This effect was reversible by rapamycin and thus dependent on mTORC1 (**Fig. 2A**). In addition, serum starvation induced the expression of PDCD4 in *Tsc1*^+/+^ and *Tsc2*^+/+^ fibroblasts (**Fig. 2B** and **D**). In contrast, PDCD4 levels were strongly reduced in either non-starved as well as starved *Tsc1*^−/−^ and *Tsc2*^−/−^ fibroblasts similar to macrophages (**Fig. 2B** and **D**). Inhibition of p38 or mTORC1 prevented anisomycin-induced degradation of PDCD4 in *Tsc1*^+/+^ and *Tsc2*^+/+^ fibroblasts (**Fig. 2C** and **D**) and in *Tsc2*^fl/fl^ BMDMs stimulated with anisomycin or LPS (**Fig. 2E** and **F**). However, BIRB796 failed to rescue PDCD4 degradation in anisomycin-stimulated *Tsc1*^−/−^ and *Tsc2*^−/−^ fibroblasts (**Fig. 2C** and **D**) and in *Tsc2*^Lyz2^ BMDMs (**Fig. 2E**). These results show that p38 controls PDCD4 expression via TSC1/TSC2. In contrast, rapamycin and the catalytic mTOR inhibitor Torin1 partially restored PDCD4 levels in anisomycin-stimulated *Tsc1*^−/−^ and *Tsc2*^−/−^ fibroblasts (**Fig. 2C** and **D**). As an ATP-competitive inhibitor, Torin1 effectively prevents both mTORC1 and mTORC2 phosphorylation [17]. Interestingly, neither rapamycin nor Torin1 restored PDCD4 in *Tsc1*^−/−^ and *Tsc2*^−/−^ cells to a level that is seen in starved *Tsc1*^+/+^ and *Tsc2*^+/+^ fibroblasts, suggesting that TSC1/TSC2 promotes basal expression of PDCD4 that is independent of mTORC1 (**Fig. 2C** and **D**). Similar results were obtained with anisomycin in BMDMs (**Fig. 2E** and **F**). However, we noticed that the inhibitors restored PDCD4 levels in LPS-supplied *Tsc2*^Lyz2^ BMDMs to a comparable level as seen in wild type-representing BMDMs. These results support the concept that anisomycin does not just simply block PDCD4 translation but actively promotes degradation of PDCD4. Previous studies have found that activation of Erk contributes to PDCD4 degradation by enhancing proteasome activity [18]. Our experiments revealed an Erk-independent manner of PDCD4 degradation in *Tsc2*^−/−^ fibroblasts since Erk expression was even reduced in the TSC2-deficient cells (**Fig. 2D**). The p90 ribosomal S6 kinases (RSKs) act downstream of Erk [19] and were shown to be promote proteasomal degradation of PDCD4 [20]. However, there was not clear association of p90RSK phosphorylation at Ser380 and PDCD4 levels in Tsc2^fl/fl^ and *Tsc2*^Lyz2^ BMDMs (Suppl. Fig. 1A). Although PDCD4 can be transcriptionally regulated [21, 22], qRT-PCR analysis of PDCD4 mRNA did not reveal significant differences between *Tsc2*^Lyz2^ and *Tsc2*^fl/fl^ BMDMs (Suppl. Fig. 1C).

### p38 activation subjects PDCD4 to proteasomal degradation

**Figure 3:**
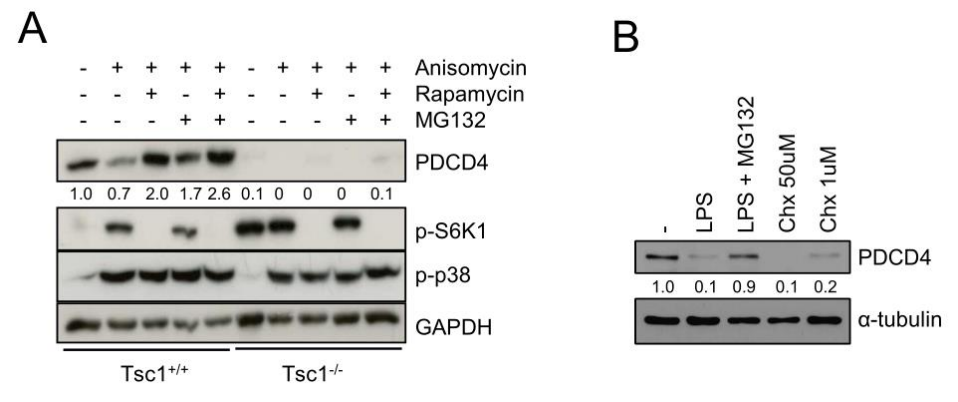
FIGURE 3: Reduction of PDCD4 is caused by p38-stimulated proteasome degradation. **(A)** *Tsc1*^*+/+*^ and *Tsc1*^*-/-*^ fibroblasts were serum-starved overnight prior to treatment with the indicated inhibitors for 90 minutes and subsequent Aniso stimulation for 4 hours. **(B)** BMDMs were treated with MG-132, LPS or cycloheximide (Chx) as indicated for 4 hours. Immunoblots were analyzed as depicted.

Finally, we tested whether p38 promotes degradation of PDCD4 via the proteasome. We noticed that the proteasome inhibitor MG-132 restored PDCD4 levels in anisomycin-treated *Tsc1*^+/+^ and *Tsc2*^+/+^ fibroblasts as well as anisomycin- and LPS-stimulated BMDMs (**Fig. 3A**, **2D–F**). However, in *Tsc1*^−/−^ and *Tsc2*^−/−^ fibroblasts as well as *Tsc2*^Lyz2^ BMDMs, MG-132 could not fully restore PDCD4 levels arguing again of an TSC1/TSC2-dependent effect that is independent of mTORC1 and proteasomal degradation (**Fig. 3A**, **2D–F**). Treating BMDMs with the p38-activating translation elongation inhibitor cycloheximide (Chx) [23, 24] confirmed that PDCD4 translation is under strong control of this MAPK (**Fig. 3B**).

## DISCUSSION

The MAPK p38α is ubiquitously expressed in most cell types and regulates diverse functions such as cell proliferation, differentiation, apoptosis, tissue repair, tumorigenesis, or inflammation [25]. Physicochemical stress signals such as heat, osmotic shock, arsenite or anisomycin result in activation of p38 [25]. p38 has been described as either tumor suppressor or oncoprotein depending on the cell type [26]. It will be interesting to evaluate whether PDCD4 contributes to the cell type-specific anti- or pro-tumorigenic functions of p38.

PI3K promotes PDCD4 degradation by mTORC1 activity in response to mitogenic signals [11]. Our data now suggests that also p38 induces degradation of PDCD4 via mTORC1 and TSC1/TSC2. We have previously shown that PI3K and p38 coordinately modulate mTORC1 signaling via TSC1/TSC2 in murine macrophages and human monocytes [13]. In agreement, LPS or anisomycin still induced partial degradation of PDCD4 in p38-deficient macrophages suggesting that PI3K and p38 also coordinately control PDCD4 degradation in macrophages (**Fig. 3C**). PDCD4 is expressed in unstressed, proliferating cells [27] and even though the heterozygous deletion of TSC2 in *Tsc2*^Lys+/-^ BMDMs creates a more proliferative macrophage type, degradation of PDCD4 by hyperactive mTORC1 in these cells outweighs healthy upregulation of PDCD4. Interestingly, rapamycin and the catalytic inhibitor Torin1, which fully blocks mTORC1 activity, did not restore PDCD4 expression in Tsc1- or Tsc2-deficient fibroblasts to wild-type levels. This indicates an additional positive regulatory role for the TSC complex on PDCD4 expression in fibroblasts independently of mTORC1. The broad PDCD4 network comprises numerous feedback loops, e. g. on PI3K/Akt [28] and dysfunctional recycling of proteins like PDCD4 by the proteasome can be compensated by autophagy [29]. PDCD4 is associated with cell cycle regulation and programmed cell death and is controlled by apoptosis inducers [9, 27, 30]. Hence, phosphorylation by protein kinases regulating survival pathways, such as casein kinase 2 (CK2), seems plausible. CK2 was already shown to interact with PDCD4 within the nucleus [31, 32] with their expression levels being inversely correlated in the tumor setting [33]. This connection would also fit into the overall picture in which PDCD4 acts pro-apoptotic [34]. Of note, CK2 can directly phosphorylate Akt to promote proliferation via mTORC1 [35]. Since PDCD4 is widely known to be regulated by microRNAs, mainly miR-21, their involvement cannot be ruled out. miR-21 is upregulated in the inflammatory and tumor-associated context [36]. However, we did not find a prominent upregulation of miR-21 in Tsc2-/- fibroblasts (data not shown).

**Figure 4:**
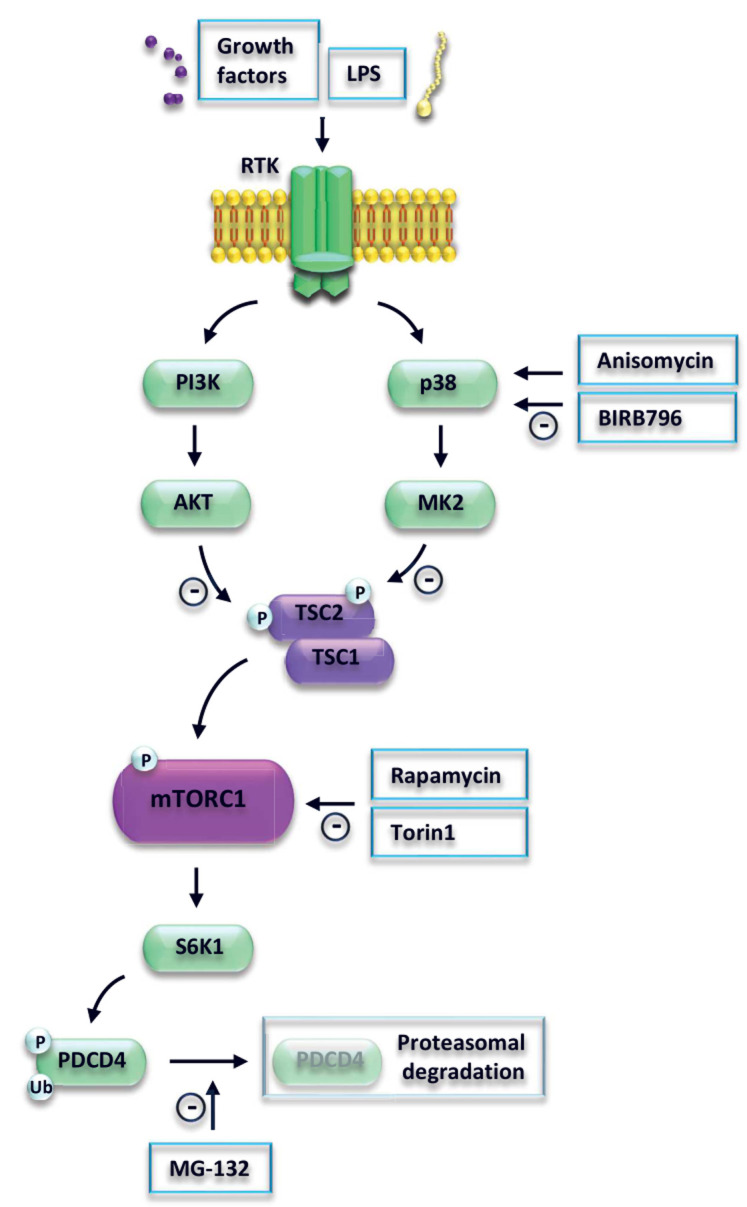
FIGURE 4: Model of p38 and mTORC1-mediated regulation of PDCD4. LPS activates Toll-like receptor 4 (TLR4) signaling. Subsequent signal transmission via phosphoinositide 3-kinase (PI3K) and mitogen-activated kinase p38 leads to inhibition of the suppressor protein tuberous sclerosis complex 2 (TSC2), followed by activation of mammalian target of rapamycin complex 1 (mTORC1). mTORC1 phosphorylates ribosomal protein S6 kinase beta-1 (S6K1), which in turn phosphorylates programmed cell death protein 4 (PDCD4). PDCD4 is ubiquitinated and degraded by the proteasome.

The precise elucidation of the upstream regulatory network that controls PDCD4 in cancer and immune cells may be important to define novel anti-cancer and anti-inflammatory strategies. In conclusion, we showed that activation of p38 promotes degradation of PDCD4 via the TSC-mTORC1 pathway (**Fig. 4**).

## MATERIALS AND METHODS

### Reagents

LPS (Lipopolysaccharide *Escherichia coli* serotype O111:B4, #LPS25), anisomycin (#A5862), rapamycin (#553211), MG-132 (#474791) and cycloheximide (#C-0943) were purchased from Sigma, BIRB796 (#5989) and Torin1 (#4247) from Tocris.

### Cell culture

Mouse embryonic fibroblasts (MEFs) were cultured in Dulbecco's Modified Eagle's Medium (DMEM) containing 4.5 g/L glucose, 2 mM L-glutamine, 100 µg/ml streptomycin, 100 U/ml penicillin and 10% heat-inactivated fetal bovine serum (FBS, Performance Plus, #10082147, Gibco). *Tsc2*^+/+^ *p53*^−/−^ and *Tsc2*^−/−^ *p53*^−/−^ as well as *Tsc1*^+/+^ and *Tsc1*^−/−^ MEFs were described previously [37]. *Mk2*^−/−^ immortalized murine macrophages stably reconstituted with MK2 or MK2K79R were kindly provided by Matthias Gaestel, Hannover, Germany. *B6;129-Tsc2*^fl/fl^ mice were kindly provided by Michael J. Gambello, Atlanta, USA [38] and were crossed to *B6.129P2-Lyz2*^tm1(cre)lfo^/J (The Jackson Laboratory) to obtain *Tsc*^fl/fl^*Lyz2*^cre/+^ (denoted *Tsc2*^Lyz2^) or *Tsc*^fl/fl^*Lyz2*^+/+^ (denoted *Tsc2*^fl/fl^) littermates. Animal care was in accord with institutional guidelines. Bone marrow-derived macrophages (BMDMs) were generated as described before [16]. BMDMs from *p38*^*f*l/fl^ and *p38*^αΔM^ mice were isolated and grown as described [39].

### Analysis of signal transduction events

BMDMs and *Mk2*^−/−^ macrophages were replated one day prior to stimulation in full medium containing 2% FBS overnight (16 h), whereas 70% confluent MEFs were completely serum-starved overnight if not stated otherwise. The cells were then treated with either 100 nM rapamycin, 200 nM BIRB796, 100 nM Torin1 or 1 µM MG-132 for 90 minutes and subsequently stimulated with 100 ng/ml LPS or 100 ng/ml anisomycin for 2 or 4 hours if not mentioned otherwise. Treatments were performed in full medium with 2% FBS, non-starved samples received 10% FBS during that time. Extract preparation and immunoblotting was done as described [40]. Antibodies were PDCD4 (clone D29C6, #9535, 1:1000 and 1:500), TSC2 (#3612 and clone D93F12, #4308, both 1:1000), p-TSC2 (Ser1254, #3616, 1:1000), p-S6 (Ser240/244, #2215 and clone D68F8, #5364, both 1:1000), p-p38 (Thr180/Tyr182, #9211, 1:1000), Erk1/2 (Thr202/Tyr204, #9102, 1:1000), p-Erk1/2 (Thr202/Tyr204, #9101, 1:1000), p-p90RSK (Ser380, #9341, 1:1000), p-H3 (Ser10, #9701, 1:1000), Vinculin (clone E1E9V, #13901, 1:1000) (all from Cell Signaling Technology) and GAPDH (#2275-PC, 1:1000, Trevigen). Molecular weight of the proteins was determined with PageRuler Prestained Protein Ladder (#26616, Thermo Scientific). With regard to quantification, data was generated either with X-ray or fluorescence detection. For X-ray detection, we applied HRP-conjugated secondary antibodies (1:10000, Bethyl Lab) and the Pierce ECL Western Blotting substrate (Thermo Scientific). Bands were visualized with Amersham Hyperfilm ECL (GE-Healthcare) and the Medical X-ray Processor 2000 system (Kodak). Fluorescence was recorded after secondary antibody incubation (IRDye IgG antibodies, 1:20000, LI-COR Biosciences) with Odyssey CLx Imaging System and analyzed with Image Studio Software (both LI-COR Biosciences). Bands were framed in unchanged manner between the samples. Normalization was performed with respect to the untreated sample of the unmodified genotype.

### mRNA expression analysis

Total RNA from BMDMs was isolated via the RNeasy Plus Mini Kit (#74134, QIAGEN) according to the manufacturer's instructions. cDNA synthesis was performed with the RevertAid RT Reverse Transcription Kit (#K1691, ThermoFisher Scientific) prior to proceeding with qRT-PCR using the GoTaq® qPCR Master Mix (#A6001, Promega). Data were acquired with a StepOnePlus Real-Time PCR System (Applied Biosystems). Relative expression was normalized to Peptidyl-prolyl cis-trans isomerase A (PPIA). The following primer pairs were used: *Pdcd4*, AGTTTTGCCCCTGGATGAGA, GCTAAGGACACTGCCAACAC*; PPIA*, TCCTGGCATCTTGTCCAT, TGCTGGTGCCATTCCT.

## AUTHOR CONTRIBUTION

CB, KK, CK, ML, NS designed and performed experiments. MH provided materials. TW designed experiments. CB, KK and TW wrote the manuscript. All authors edited and approved the final draft.

## SUPPLEMENTAL MATERIAL

Click here for supplemental data file.

All supplemental data for this article are available online at www.cell-stress.com/researcharticles/2021a-braun-cell-stress/.

## Abbreviations:

BMDM: – bone marrow-derived macrophage;
CK2: – casein kinase 2;
LPS: – lipopolysaccharide;
MAPK: – mitogen-activated kinase;
MK2: – MAP kinase-activated protein kinase 2;
mTORC1: – mammalian target of rapamycin complex 1;
PDCD4: – programmed cell death protein 4;
PI3K: – phosphoinositide 3-kinase;
RSK: – ribosomal S6 kinase;
TSC: – tuberous sclerosis complex.
