# Supplementary material for: p38 regulates the tumor suppressor PDCD4 via the TSC-mTORC1 pathway
Source: Cell Stress. 2021 Nov 23;5(12):176–82. doi: 10.15698/cst2021.12.260 (PMC8645265; doi:10.15698/cst2021.12.260)
Supplement: Supplementary file 1 [file ces-05-176-s01.pdf]

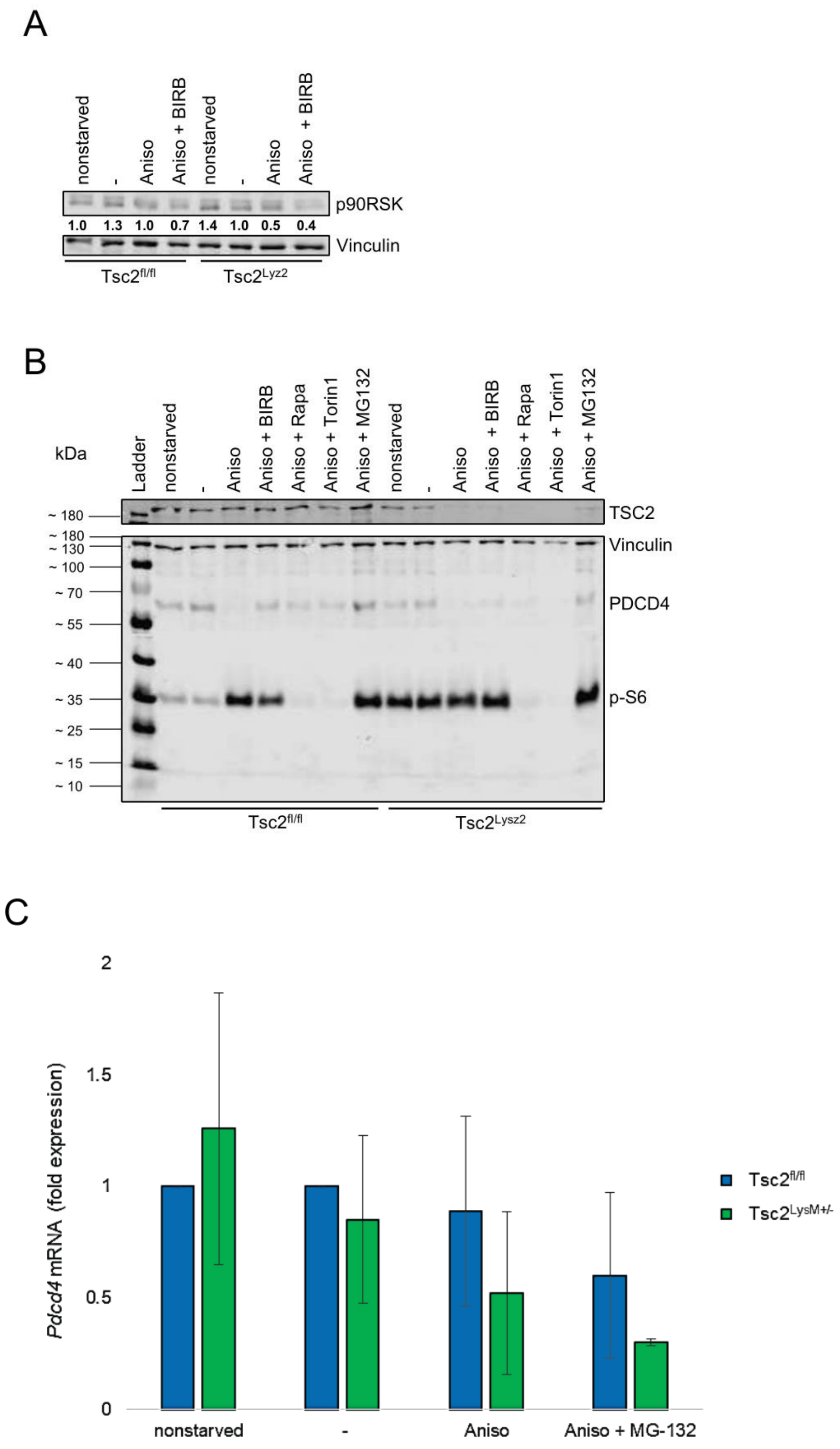

Supplemental Figure 1. (A) BMDMs from *Tsc2*$^{fl/fl}$ and *Tsc2*$^{Lyz2-}$ mice were pretreated with BIRB796 and stimulated with anisomycin as indicated. Immunoblots were analyzed as depicted. **(B)** Representative uncut images with molecular weight marker for immunoblotting experiments. **(C)** *Tsc2*$^{fl/fl}$ and *Tsc2*^{Lyz2} BMDMs were pretreated with MG-132 as depicted and then stimulated with anisomycin for 2h. $2^{-\Delta\Delta Ct}$ values show *Pdcd4* mRNA expression levels normalized to *PPIA* expression and are representative of two independent experiments.
